# Development of a novel copper metabolism-related risk model to predict prognosis and tumor microenvironment of patients with stomach adenocarcinoma

**DOI:** 10.3389/fphar.2023.1185418

**Published:** 2023-05-22

**Authors:** Dongjie Sun, Haiying Zhang, Chi Zhang

**Affiliations:** ^1^ Department of Translational Medicine, The First Hospital of Jilin University, Changchun, China; ^2^ College of Basic Medical Sciences, Jilin University, Changchun, China; ^3^ Key Laboratory of Pathobiology, Ministry of Education, College of Basic Medical Sciences, Jilin University, Changchun, China; ^4^ Department of Anesthesiology, The First Hospital of Jilin University, Changchun, China

**Keywords:** copper, copper metabolism-related genes, prognosis, immunotherapy, biomarkers, single-cell RNA sequencing, IMvigor210

## Abstract

**Background:** Stomach adenocarcinoma (STAD) is the fourth highest cause of cancer mortality worldwide. Alterations in copper metabolism are closely linked to cancer genesis and progression. We aim to identify the prognostic value of copper metabolism-related genes (CMRGs) in STAD and the characteristic of the tumor immune microenvironment (TIME) of the CMRG risk model.

**Methods:** CMRGs were investigated in the STAD cohort from The Cancer Genome Atlas (TCGA) database. Then, the hub CMRGs were screened out with LASSO Cox regression, followed by the establishment of a risk model and validated by GSE84437 from the Expression Omnibus (GEO) database. The hub CMRGs were then utilized to create a nomogram. TMB (tumor mutation burden) and immune cell infiltration were investigated. To validate CMRGs in immunotherapy response prediction, immunophenoscore (IPS) and IMvigor210 cohort were employed. Finally, data from single-cell RNA sequencing (scRNA-seq) was utilized to depict the properties of the hub CMRGs.

**Results:** There were 75 differentially expressed CMRGs identified, 6 of which were linked with OS. 5 hub CMRGs were selected by LASSO regression, followed by construction of the CMRG risk model. High-risk patients had a shorter life expectancy than those low-risk. The risk score independently predicted STAD survival through univariate and multivariate Cox regression analyses, with ROC calculation generating the highest results. This risk model was linked to immunocyte infiltration and showed a good prediction performance for STAD patients’ survival. Furthermore, the high-risk group had lower TMB and somatic mutation counters and higher TIDE scores, but the low-risk group had greater IPS-PD-1 and IPS-CTLA4 immunotherapy prediction, indicating a higher immune checkpoint inhibitors (ICIs) response, which was corroborated by the IMvigor210 cohort. Furthermore, those with low and high risk showed differential susceptibility to anticancer drugs. Based on CMRGs, two subclusters were identified. Cluster 2 patients had superior clinical results. Finally, the copper metabolism-related TIME of STAD was concentrated in endothelium, fibroblasts, and macrophages.

**Conclusion:** CMRG is a promising biomarker of prognosis for patients with STAD and can be used as a guide for immunotherapy.

## Introduction

The most common and malignant subtype of gastric cancer is STAD (Correa, 2013; Smyth et al., 2020; Sung et al., 2021). Surgery is the primary therapy for STAD; however, Patients are often diagnosed at an advanced stage, with poor prognosis and a less than 10% 5-year survival rate. (Tan, 2019; Smyth et al., 2020). The prognosis of STAD varies depending on the diagnosis and response to treatment (Roukos, 2000). Early diagnosis and treatment may improve the likelihood of success. However, the molecular processes of STAD are still unknown. Given the limits of STAD therapy options, it is critical to conduct more research to uncover novel prognostic indicators and possible therapeutic targets for STAD.

Copper metabolism involves the absorption, distribution, sequestration, and excretion of copper and is one of the trace elements required by living systems (Chen et al., 2020). Copper is the third most prevalent transition metal in humans and is required for the growth and reproduction of all eukaryotes (Andreini et al., 2008). Copper homeostasis is related to cell proliferation, angiogenesis, and metastasis (Fukai et al., 2018; ATP, 2023), abnormal copper metabolism can lead to various diseases such as Wilson’s disease, Menkes’ disease, and idiopathic copper toxicosis (Shobha Devi et al., 2018; Tasić et al., 2022). Copper ions directly bind to tricarboxylic acid cycle components, triggering lipid-acylated protein aggregation and cell death. (Kim et al., 2008). Recently, copper has been shown to regulate the expression of programmed death ligand 1 (PDL1), a transmembrane protein regulated on the surface of some cancer cells that allows immune evasion (Voli et al., 2020). Furthermore, the anti-tumor effects of the immune response depend on effective mitochondrial function, and copper deficiency inhibits the immune response (Shanbhag et al., 2021). Therefore, the risk model of CMRGs could anticipate STAD prognosis and treatment response.

Here we developed a risk model based on five five-pivotal CMRGs, including CP, F5, LOX, S100A12, and SNCG, to predict prognosis, immune microenvironment, and immunotherapy in STAD patients. Our models showed a good prediction of survival in STAD patients, as well as immunotherapy and drug sensitivity to provide personalized treatment for STAD patients.

## Materials and methods

### Data collection

RNA-seq data and clinical information for STAD patients were downloaded from the TCGA portal (375 STAD and 32 normal tissues). The GEO database was used to get the GSE84437 dataset, which included 433 STAD sequencing data, and the GSE167297 single-cell RNA sequencing dataset.

### CMRG identification

CMRGs were referenced from previous literature (Chang et al., 2022). Differentially expressed CMRGs were selected with |log_2_FC| ≥ 0.585 & p.adj <0.05 by the “limma” package (Ritchie et al., 2015). OS-related CMRGs were recognized by univariate Cox regression (van Dijk et al., 2008). Hub CRMGs were screened out by LASSO Cox regression using the “glmnet” package.

### Relationship between risk model and clinical characteristics

The connection between risk ratings and clinical variables was investigated using chi-square testing. Overall survival (OS) was assessed for risk groups and subgroups with different clinical characteristics using KM curves from the “survminer” package.

### Nomogram construction

We developed a nomogram for OS based on conventional clinical traits and CMRGS to estimate the prognosis of STAD patients. A nomogram was created using the “rms” software. To assess the model’s accuracy and establish the prediction value, ROC and calibration curves were employed.

### TMB analysis

Somatic mutation data was analyzed for STAD patients in high- and low-risk with the “maftools” package (Ng et al., 2018).

### Genome enrichment analysis (GSEA)

GSEA was performed with the “GSVA” package to define biological functions (Subramanian et al., 2005). The threshold was set as *p* < 0.05 & FDR <0.25.

### TICs landscape

Single gene set enrichment analysis (ssGSEA) was performed on tumor-infiltrated immune cells (TICs) (Hänzelmann et al., 2013). Tumor purity and immune score were then assessed using the “ESTIMATE” package. Spearman’s correlation analysis was used to determine the relationship between risk score and TICs.

### TIDE assessment

T-cell dysfunction, rejection, and checkpoint inhibitor responsiveness are evaluated using the Tumor Immune Dysfunction and Rejection (TIDE) technique. A greater likelihood of anti-tumor immunosuppression is indicated by higher TIDE scores.

### Predicting response to immunotherapy

IPS scores of STAD patients and the imvigor210 cohort were performed for immunotherapy response prediction. IPS scores of immunotherapy response against CTLA-4 and PD-1 of STAD patients were collected (Charoentong et al., 2017). The patients in the Imvigor210 validation cohort had locally progressed or metastatic urothelial carcinoma (Mariathasan et al., 2018).

### Predicting drug sensitivity

The half-maximal inhibitor concentration (IC50) of anticancer medications was provided to various subgroups of patients using the “oncoPredict” package. The threshold was defined as *p* < 0.001.

### Consensus clustering

The k-means approach was used to uncover different patient patterns linked to the expression of CMRGs using consensus clustering. The number and stability of clusters were established using the consensus clustering methods, which were implemented in the “ConsensuClusterPlus” package. To ensure the reliability of our categorization, we repeated it 1,000 times.

### Real-time quantitative PCR analysis

To examine the expression of the 5 hub CMRGs, total RNA (1 μg) of NGEC, SGC-7901, and BGC-823 cell lines were isolated using the TRIzol reagent (Invitrogen, United States), and first-strand complementary DNA was synthesized using SuperScript III Reverse Transcriptase (Invitrogen) and oligo-dT (Promega, United States), according to the manufacturer’s instructions. qPCR was performed using SYBR green (Sigma). The 2^−ΔΔCT^ calculation method was performed. Primer sequences: CP: forward 5′- AAA​TGA​AGA​CAC​CAA​ATC​TGG​C-3′, reverse 5′-ACA​AAG​TTG​TAT​GCT​TCC​AGT​C-3’; F5: forward 5′- TAT​CAT​GGA​CAG​AGA​CTG​TAG​G-3′, reverse 5′-AAC​TCT​GAA​GCC​TTG​ATC​TG-3’; LOX: forward 5′-CAA​GGG​ACA​TCA​GAT​TTC​TTA​CC-3′, reverse 5′-CCA​TAC​TGT​GGT​AAT​GTT​GAT​GAC-3’; S100A12: forward 5′-AAA​GGA​GCT​TGC​AAA​CAC​C-3′, reverse 5′-ATT​AGC​ATC​CAG​GCC​TTG​G-3’; SNCG: forward 5′-TGT​ATG​TGG​GAG​CCA​AGA​C-3′, reverse 5′-CAG​ATG​GCC​TCA​AGT​CCT​C-3’; GAPDH: forward 5′-TCA​AGA​TCA​TCA​GCA​ATG​CC-3′, reverse 5′- CGA​TAC​CAA​AGT​TGT​CAT​GGA-3’.

### Human Protein Atlas (HPA) database and immunohistochemistry (IHC) verification

The protein expressions of risk CMRGs in STAD tissues were examined using the Human Protein Atlas (HPA) database (https://www.proteinatlas.org/), which aspires to develop a human proteome-wide map using integrated omics technologies. IHC was used to evaluate the protein expression patterns in STAD samples collected from the China-Japan Union Hospital of Jilin University for CMRGs that were not included in the HPA database. The ethics committee of the China-Japan Union Hospital of Jilin University accepted the study (NO:2023-KYYS-023). The paraffin-embedded STAD tissues were IHC stained after routine embedding, sectioning, dewaxing, and rehydration methods. Briefly, slices were treated overnight at 4°C with primary antibodies (anti-LOX, 1:200, Abclonal, A11504; anti-FV, 1:200, Affinity, DF8265) before being incubated for 1 h at 37°C with biotinylated goat anti-rabbit IgG secondary antibody. The expression of LOX and FV was then visualized using diaminobenzidine tetrachloride (DAB) staining.

### Single-cell RNA-seq analysis

Tumor Immune Single-cell Hub (http://tisch.comp-genomics.org/home/) was used to do single-cell RNA-seq analysis, with the UMAP approach used to decrease dimensionality and display clustering findings. UMAP distribution pictures were also used to visualize the mRNA expression of distinct cells.

### Statistical analysis

All statistical analyses were performed using R. The Wilcoxon test was performed to compare the differences between the two groups. For the correlation analysis, Spearman’s rank correlation was used. *p* < 0.05 was deemed statistically significant.

## Results

### Development and evaluation of the CMRG risk model in STAD

**FIGURE 1:**
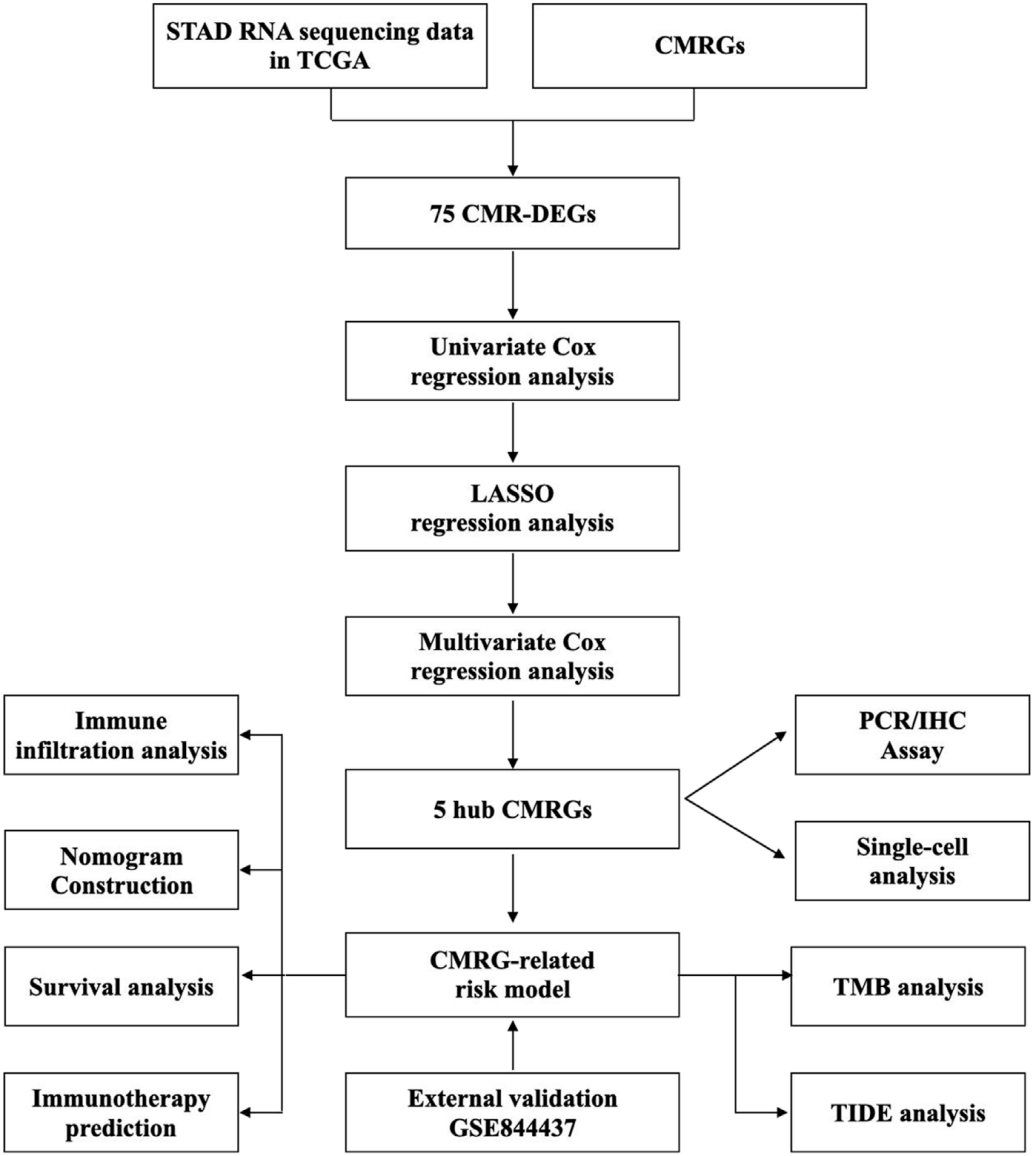
Workflow of the study.

**FIGURE 2:**
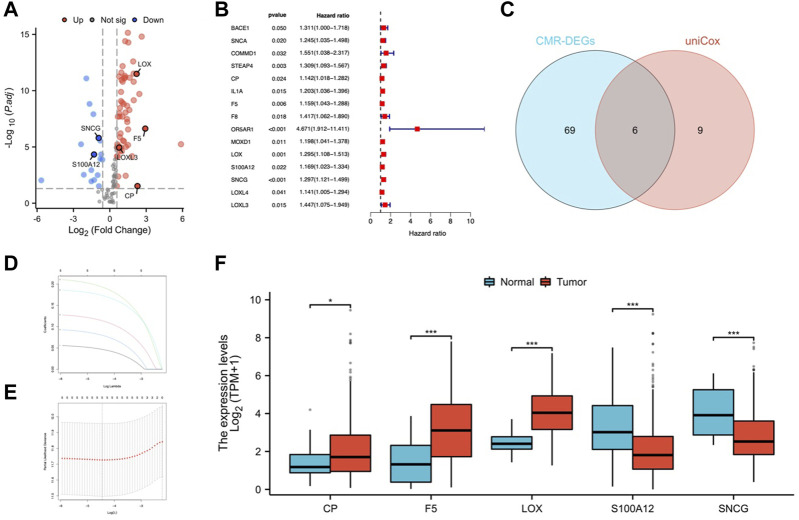
Identification of CMRGs in STAD. **(A)** Volcano map of CMRGs with different expressions. **(B)** Predictive value of CMRG. **(C)** Venn diagram of selected prognostic CMR-DEGs. **(D, E)** Lasso Cox regression analysis of 6 prognostic CMR-DEGs. **(F)** mRNA expression of CP, F5, LOX, S100A12 and SNCG in STAD and normal tissues. *p < 0.05; **p < 0.01; ***p < 0.001.

Figure 1 depicted the flow of the study. 75 differentially expressed CMRGs were identified (Figure 2A; Supplementary Figure S1), including 58 upregulated and 17 downregulated DEGs. 15 prognostic CMRGs were screened out (*p* < 0.05; Figure 2B; Supplementary Table S1 **)**. Subsequently, six common genes were identified using Venn diagrams (Figure 2C). Finally, Lasso Cox regression further screened out 5 hub CMRGs (Figures 2D, E), where CP, F5, and LOX were elevated in STAD tissues, while S100A12 and SNCG were downregulated compared to normal tissues (Figure 2F). Finally, multivariate Cox regression determined corresponding coefficients and risk score for each patient was calculated: CP exp * (0.04668) + F5 exp * (0.1139) + LOX exp * (0.1931) + S100A12 exp * (0.07975) + SNCG exp * (0.1733).

### Prognostic value and validation for risk model

**FIGURE 3:**
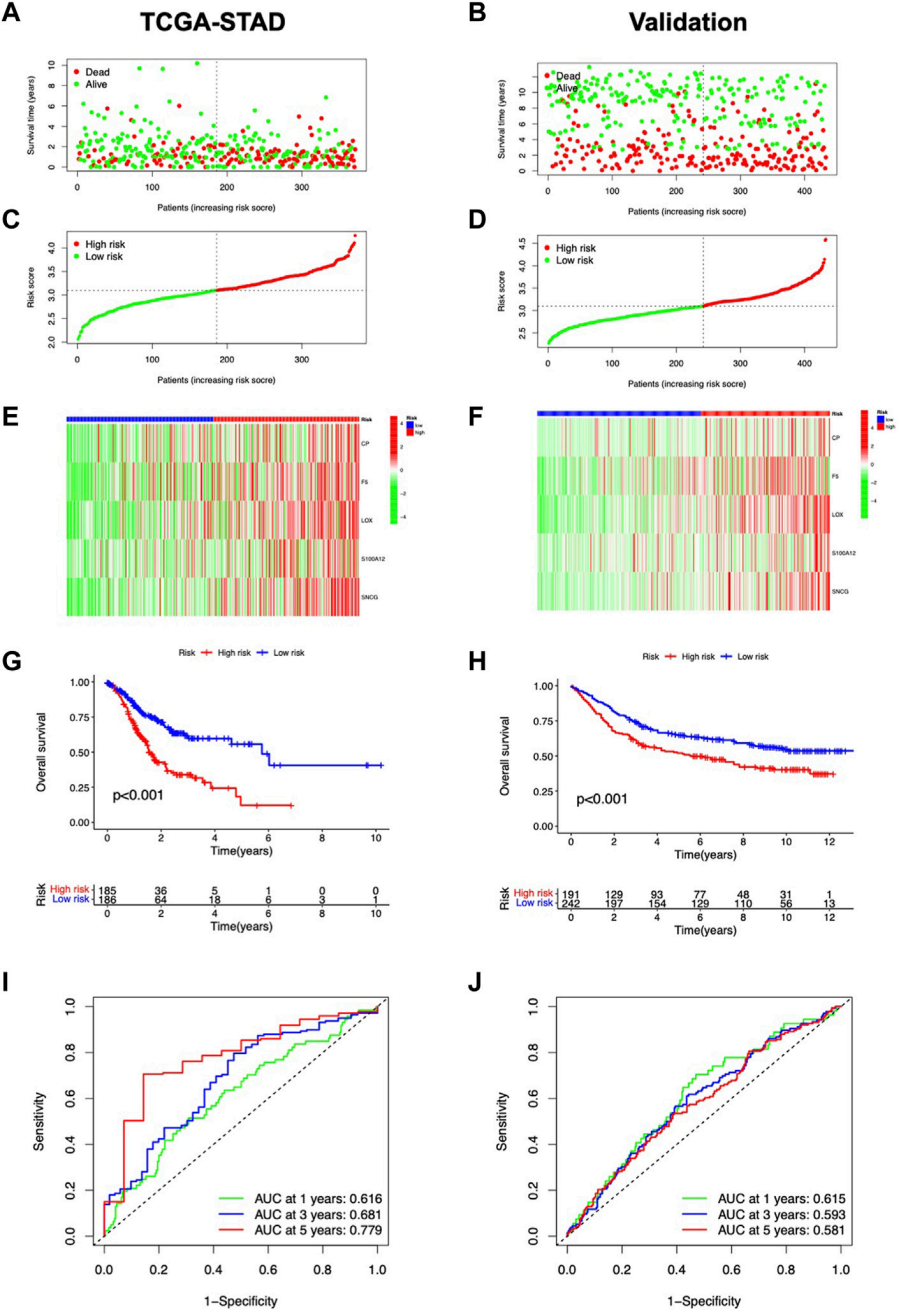
Predictive value assessment of the risk model. Distribution of overall survival status **(A)**, and risk scores **(B)**. **(C)** Heatmap of CMRG expression. **(D)** Kaplan-Meier curves for OS in the TCGA-STAD cohort. **(E)** ROC curves for OS prediction. The distribution of overall survival status **(F)**, and risk score **(G)**. **(H)** CMRG expression in the validation cohort. **(I)** Kaplan-Meier survival curves for OS in the validation cohort. **(J)** Validation of ROC curves for OS prediction in the validation cohort.

Based on the medial risk score, STAD patients were grouped into high-risk and low-risk categories. Figures 3A, B shows the distribution of survival status and risk score in the risk model. The expression of CP, F5, LOX, S100A12, and SNCG was shown by heatmap (Figure 3C). Patients of low-risk outlived the high risk (Figure 3D). The AUCs for OS at 1, 3, and 5 years were 0.616, 0.681, and 0.779 (Figure 3E). The GSE84437 cohort was used for model validation (Figures 3F–J). OS in the validation dataset showed that the low-risk group had better clinical outcomes (Figure 3I).

### Independent analysis and nomogram construction

**FIGURE 4:**
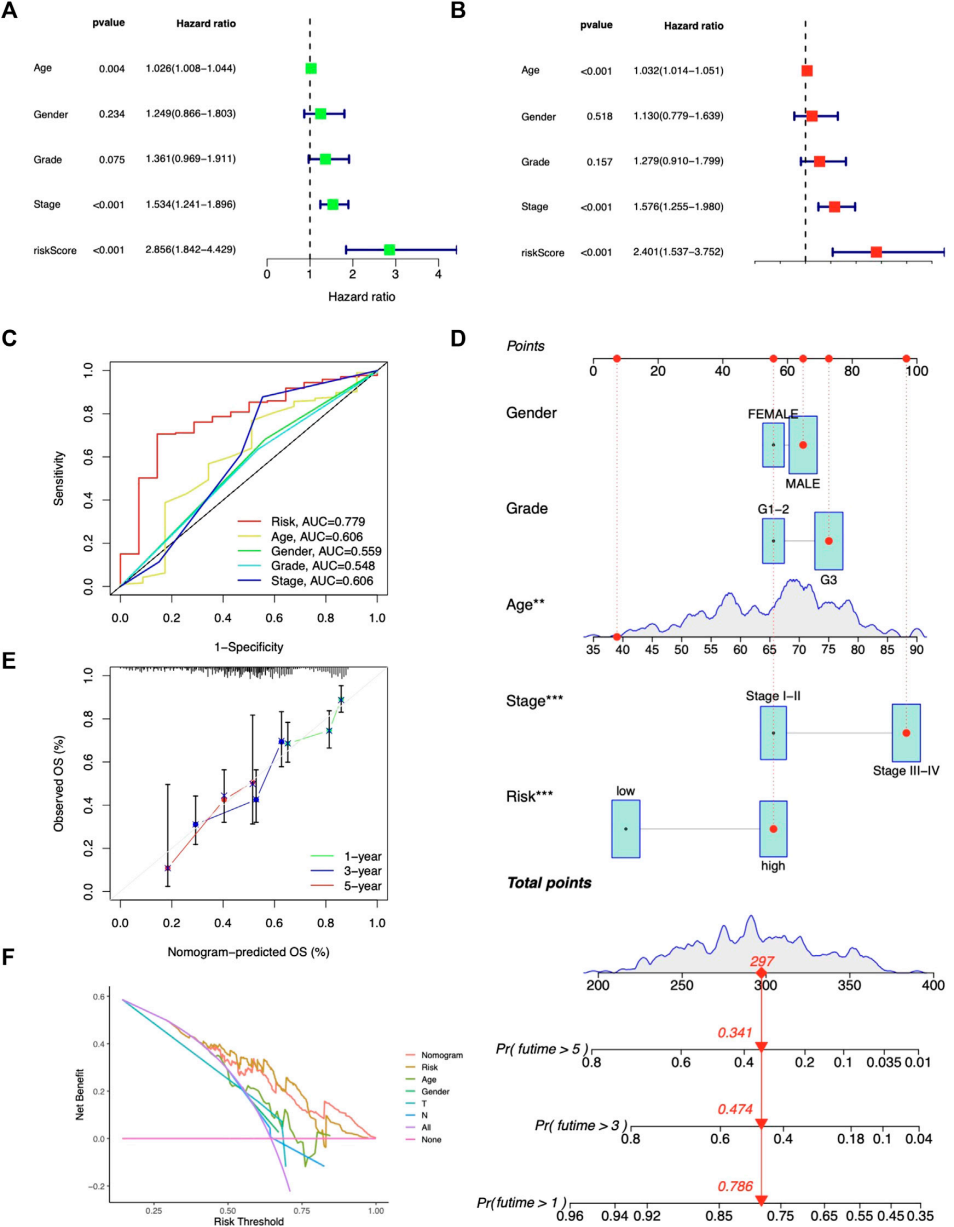
Predictive value of the risk model. Independent prediction analysis by univariate **(A)** and multivariate Cox regression **(B)**. **(C)** Predictive accuracy of the risk model in terms of age, gender, grade, and stage. **(D)** Nomogram for OS predictions. **(E)** Calibration curves for OS predictions. **(F)** DCA curves for 5-year clinical predictions.

The risk score was shown related to OS (HR = 2.856, 95% CI 1.842-4.429) (Figure 4A); in multivariate Cox regression, the risk score remained an independent prognostic factor (HR = 2.401, 95% CI 1.537-3.752) (Figure 4B), both of which had higher HR than other clinical factors. The AUC of the risk model reached 0.779 (Figure 4C). Then, based on age, gender, stage, grade, and risk, nomograms were built to predict patients’ OS (Figure 4D). The predicted OS values were consistent with the actual, according to calibration curves (Figure 4E). DCA curves showed that nomograms and risk scores predicted OS better than other factors (Figure 4F **)**. Additionally, a nomogram using risk score and clinicopathological factors for PFS prediction of STAD patients (Supplementary Figure S2). Calibration curves also showed significant agreement (Supplementary Figures S2A, B). All these results consistently suggest that CMRG risk characteristics are independent predictors of STAD.

### Clinical characteristics in the risk model

**FIGURE 5:**
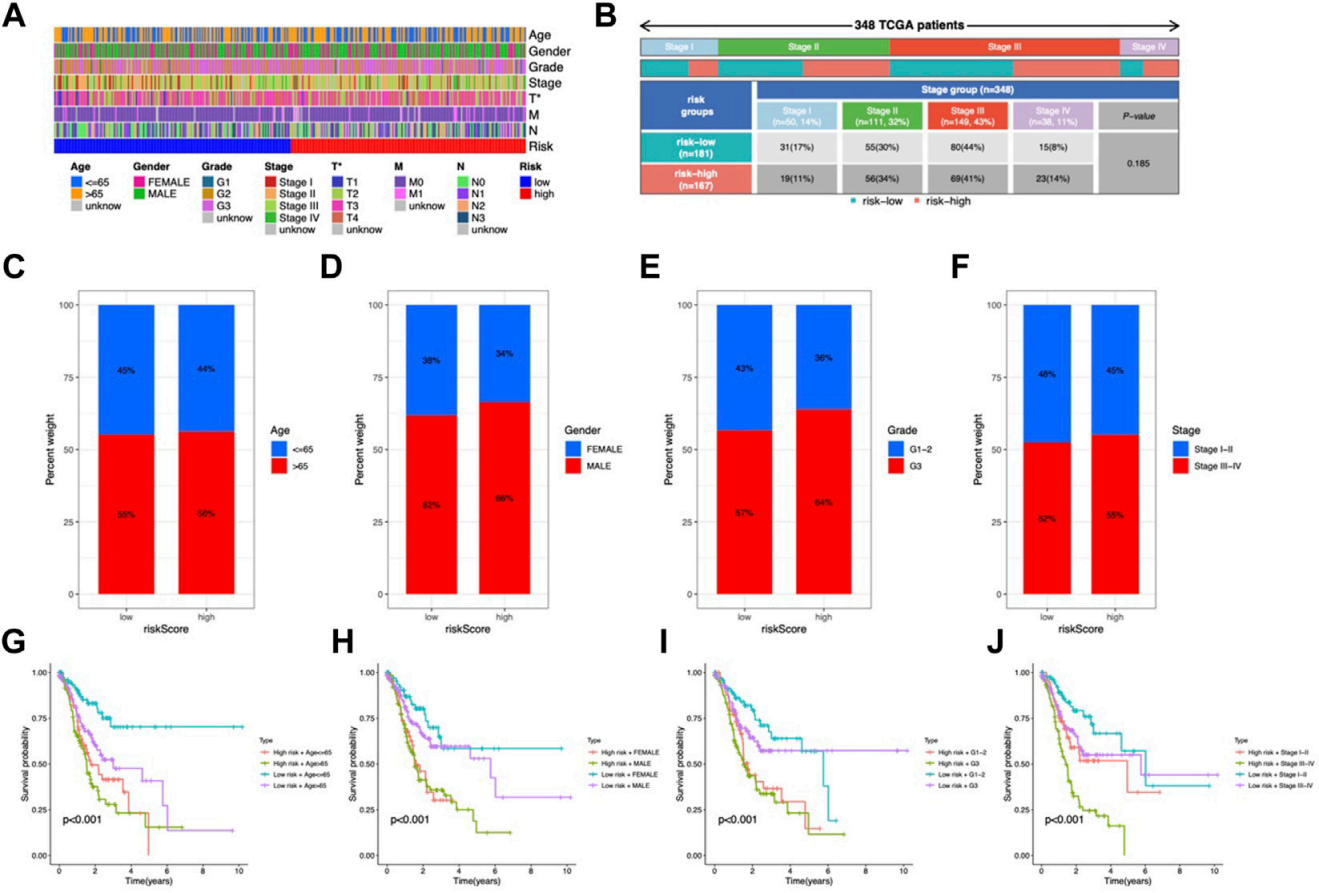
Clinicopathological characteristics in the risk model. **(A)** Heatmap of clinical features and risk score distribution. **(B)** Incidence of high-risk and low-risk tumor stages. The proportion of patients by age **(C)**, gender **(D)**, grade **(E)**, and stage **(F)**. **(G–J)** OS analysis of subgroup.

In the clinical heatmap, the T stage varied in risk groups (Figure 5A **)**. More stage III and IV individuals were observed in the high-risk category (Figure 5B). Patients over 65 years old, male, G3, and stage III-IV accounted for a higher proportion in the high-risk group (Figures 5C–F). Then, the STAD cohort was grouped according to age (≤65 or >65 years), gender (female or male), grade (G1-2 or G3), and stage (stage I-II or III-IV). As shown in Figures 5G–J, high-risk patients had a worse prognosis, with patients aged >65 years, stage G3 and stage III-IV having a worse prognosis in their respective groups. Based on these results, The CMRG risk model is a reliable predictor that may be used to evaluate the prognosis of STAD patients.

### Functional assessment

**FIGURE 6:**
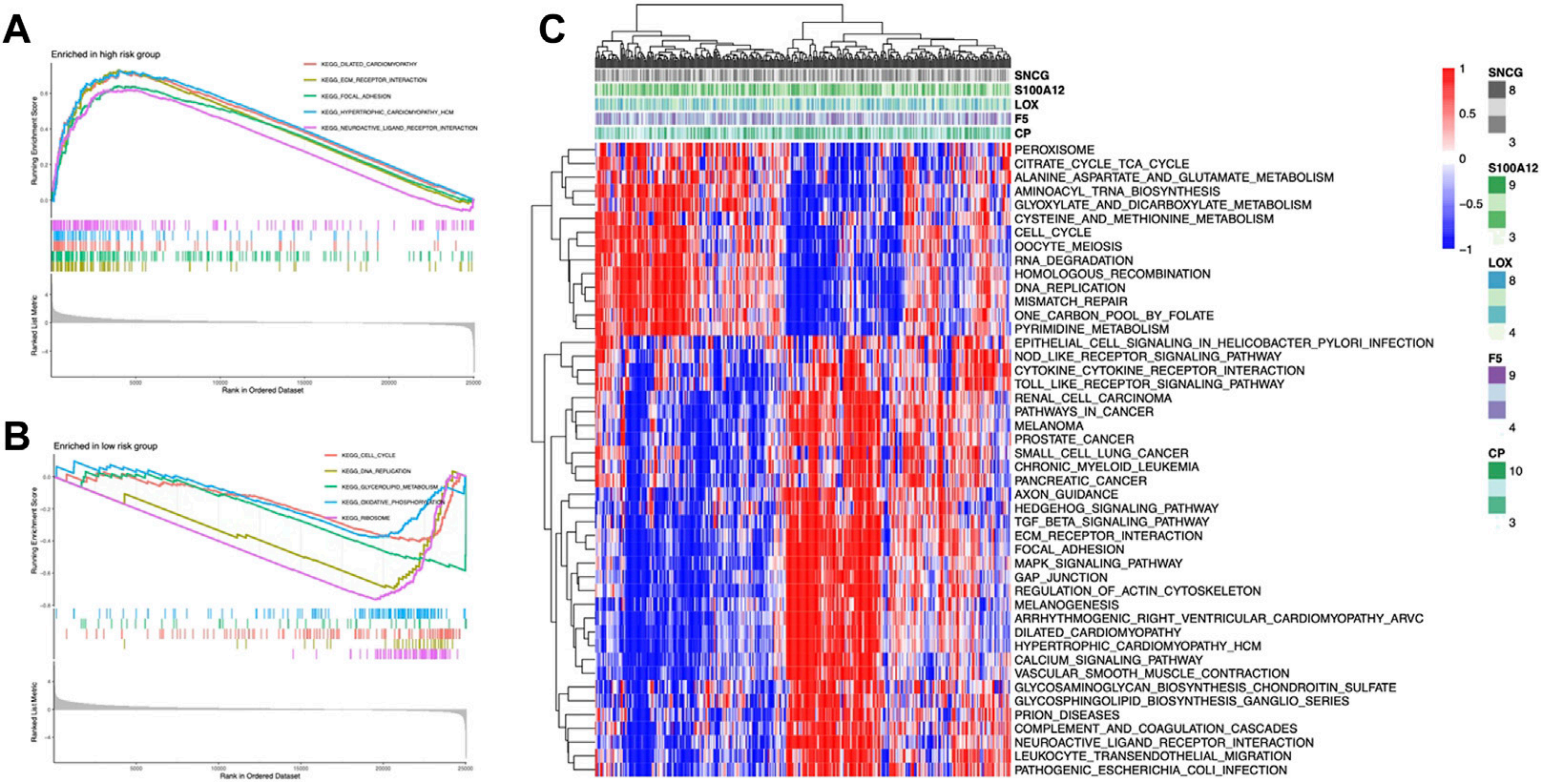
CMRG enrichment analysis. GSEA of KEGG pathways of the high-risk group **(A)** and low-risk group **(B)**. **(C)** Heatmap of pathways enrichment related to CMRGs.

We explored the functional pathways in the model using GSEA analysis and found that ECM receptor interactions, dilated capillaries, and focal adhesion pathways were enriched in the high-risk group (Figure 6A); the low-risk group had higher enrichment of the oxidative phosphorylation, DNA replication, glycolipid metabolism, and cell cycle (Figure 6B). Additionally, GSVA demonstrated that a total of 46 pathways, including the peroxisome, the TCA cycle, and some immune-related pathways including the TGF-β pathway and MAPK pathway, were considerably enriched in the risk model (Figure 6C). Most of the enriched pathways showed a positive correlation with LOX (Supplementary Figure S3).

### TMB analysis of the risk model

**FIGURE 7:**
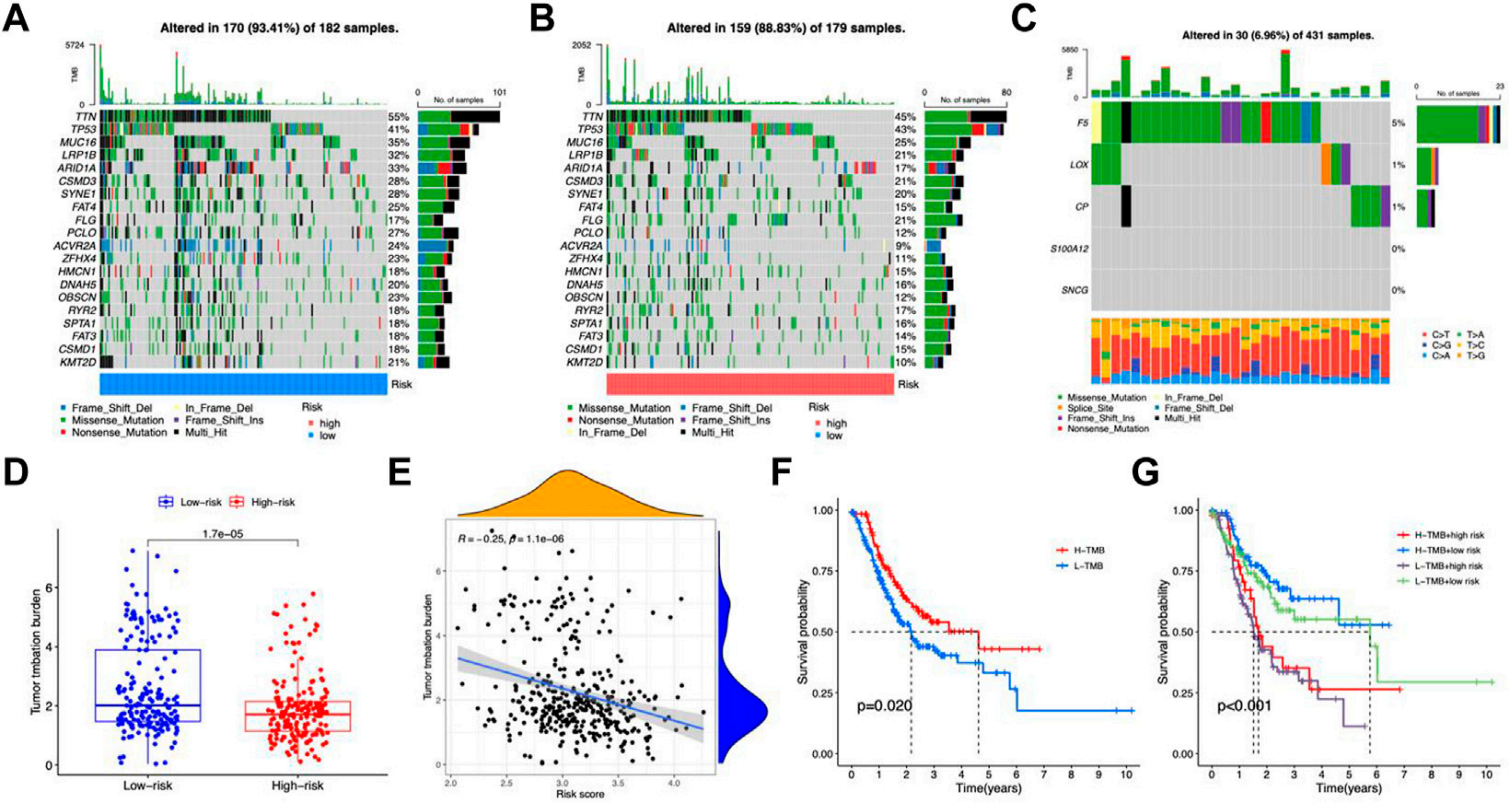
TMB analysis. Oncoprint of mutations in the high-risk group **(A)** and low-risk group **(B)**. **(C)** Gene mutations of CMRGs. **(D)** Differences in TMB between the two risk groups. **(E)** Scatter plot of correlation between risk score and TMB. **(F)** Kaplan-Meier curves for the high- and low-TMB groups. **(G)** Kaplan-Meier curves for patients with different TMB and risk scores.

To investigate the possible association between somatic mutations and risk patterns, we analyzed the distribution of total TMBs and mutations. The mutation frequency was greater in the group with the lower risk and the salient features of the mutated genes indicated that TTN (55% vs. 45%), TP53 (41% vs. 43%), and MUC16 (35% vs. 25%) ranked in the top three somatic mutations in both risk groups (Figures 7A, B). We then tested risk models for mutations in five genes. This showed that F5, LOX, and CP were mutated in a higher number of samples, while no mutations were observed in S100A12 and SNCG SNV (Figure 7C). Among the five genes, only a very small number of samples were found to have gain/loss of CNV (Supplementary Figure S4A). TMB was higher in the low-risk group (Figure 7D). TMB showed a negative correlation with risk score (R = −0.25, *p* < 0.001, Figure 7E). Patients with H-TMB had significantly longer OS than those with L-TMB (Figure 7F). The L-TMB with a high-risk score had the worst OS (Figure 7G).

### Immune feature in the risk model

**FIGURE 8:**
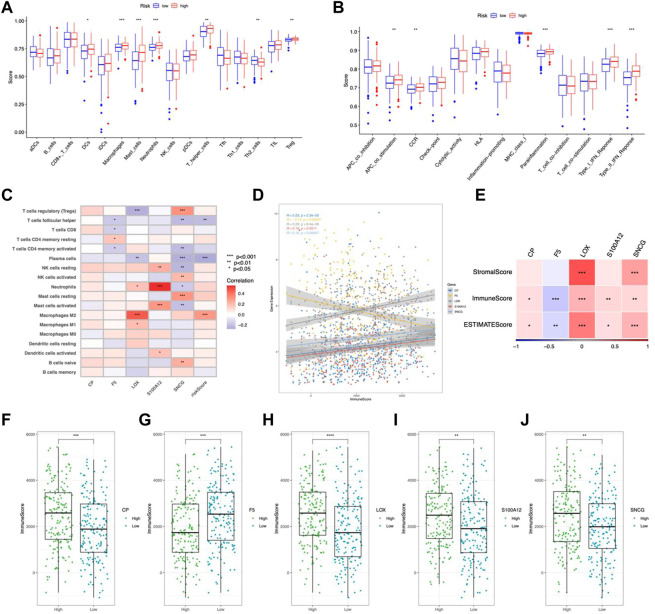
Immune feature in the risk model. **(A)** TICs distribution. **(B)** Immune function scores comparison. **(C)** Correlation between TICs and risk scores. **(D)** Correlation between CMRGs and immune scores. **(E)** Correlation between CRMG and ESTIMATE scores and stromal scores. Immune scores were correlated with the expression of CP **(F)**, F5 **(G)**, LOX **(H)**, S100A12 **(I)**, and SNCG **(J)**. *p < 0.05; **p < 0.01; ***p < 0.001.

**FIGURE 9:**
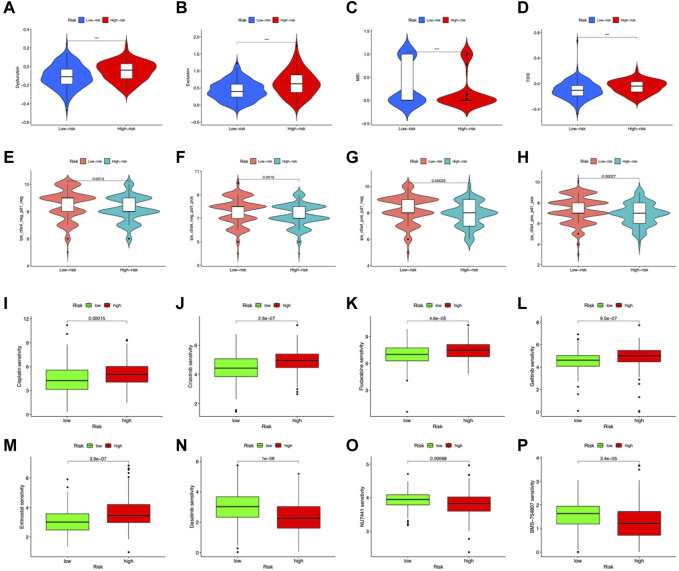
Predicted response to immunotherapy. Analysis of **(A)** dysfunction, **(B)** exclusion, **(C)** MSI, and **(D)** TIDE scores between the high-risk and low-risk groups. Comparison of IPS in the two groups with CTLA4_negative_/PD-1_negative_ **(E)**, CTLA4_negative_/PD-1_positive_ **(F)** CTLA4 _positive_/PD-1_negative_ **(G)** CTLA4_positive_/PD-1_positive_ **(H)**. Sensitive drugs in the low-risk group **(I–M)** and high-risk group **(N–P)**.

In the high-risk group, DCs, macrophages, mast cells, neutrophils, and Tregs were more abundant (Figure 8A). Analysis of immune function showed higher APC costimulation, CCR, and IFN responses in the high-risk group (Figure 8B), indicating an active TME status in patients with high risk. Additionally, the risk score was positively connected with M2 macrophages, whereas LOX and S100A12 exhibited substantial positive correlations with neutrophils and M2 macrophages, respectively (Figure 8C). CP, LOX, S100A12, and SNCG positively connected with the immune score and F5 negatively correlated (Figures 8D–J). TIDE analysis showed higher dysfunction, rejection, and TIDE scores but lower MSI scores in the high-risk group, indicating a higher possibility of immune escape (Figures 9A–D).

### Prediction of response to immunotherapy based on the CMRG risk model

IPS, IPS-CTLA4, IPS-PD-1, and IPS-PD-1 + CTLA4 scores were used to examine the outcomes of ICIs treatment. The low-risk group had higher IPS scores in all groups (Figures 9E–H). Indicating a better outcome for ICI immunotherapies. In addition, we used the IMvigor210 cohort to validate risk model-based prediction of immunotherapy response. For 298 samples, risk ratings were determined and partitioned into two groups. The prognosis was better in the low-risk group (*p* = 0.0016, Figure 9I). In patients with binary responses and stage I-II patients, the CR/PR ratio was higher in the low-risk group (Figure 9J). These results suggest that low-risk patients of the CMRG model potentially benefit more from immunotherapy.

### Chemotherapy predictions

**FIGURE 10:**
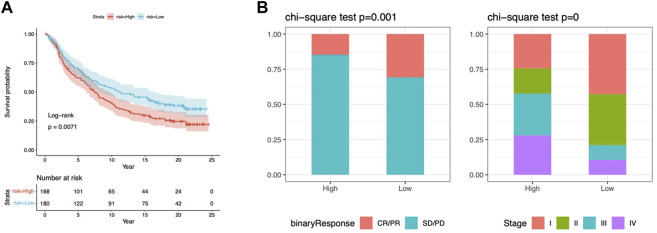
Response to chemotherapy in the risk model. **(A)** Kaplan-Meier analysis of OS in the IMvigor210 cohort. **(B)** The proportion of immunotherapy responses and stages in risk groups. cr, complete response; pr, partial response; sd, stable disease, pd, disease progression.

Regarding potential therapeutic use, we analyzed drug sensitivities in both risk categories. Overall, Cisplatin, Crizotinib, Gefitinib, Fludarabine, and Entinostat showed significant sensitivity in low-risk patients (Figures 10A–E). The high-risk group showed high sensitivity to Dasatinib, NU7441, and BMS-754807 (Figures 10F–H). In clinical trials, low-risk individuals were more susceptible to first-line medicines (Koizumi et al., 2008; Walker et al., 2009; Shaw et al., 2020; Mok et al., 2021). Furthermore, as a synthetic benzamide derivative class I histone deacetylase inhibitor, Entinostat has been examined in Phase I and II studies and is typically well tolerated in patients with advanced malignancies (Connolly et al., 2017). Dasatinib is being used to treat chronic myeloid leukemia in high-risk patients (Talpaz et al., 2018). In pancreatic cancer, BMS-754807, a small-molecule inhibitor of the insulin-like growth factor-1 receptor/insulin receptor, improves gemcitabine responsiveness (Awasthi et al., 2012). NU7441 reduced NSCLC cell proliferation and increased chemosensitization to topoisomerase inhibitors by preventing DNA repair (Yanai et al., 2017). These findings point to a possible relationship between risk score and chemotherapeutic drugs for individualized treatment.

### Consensus clustering analysis of CMRG-based subgroups

**FIGURE 11:**
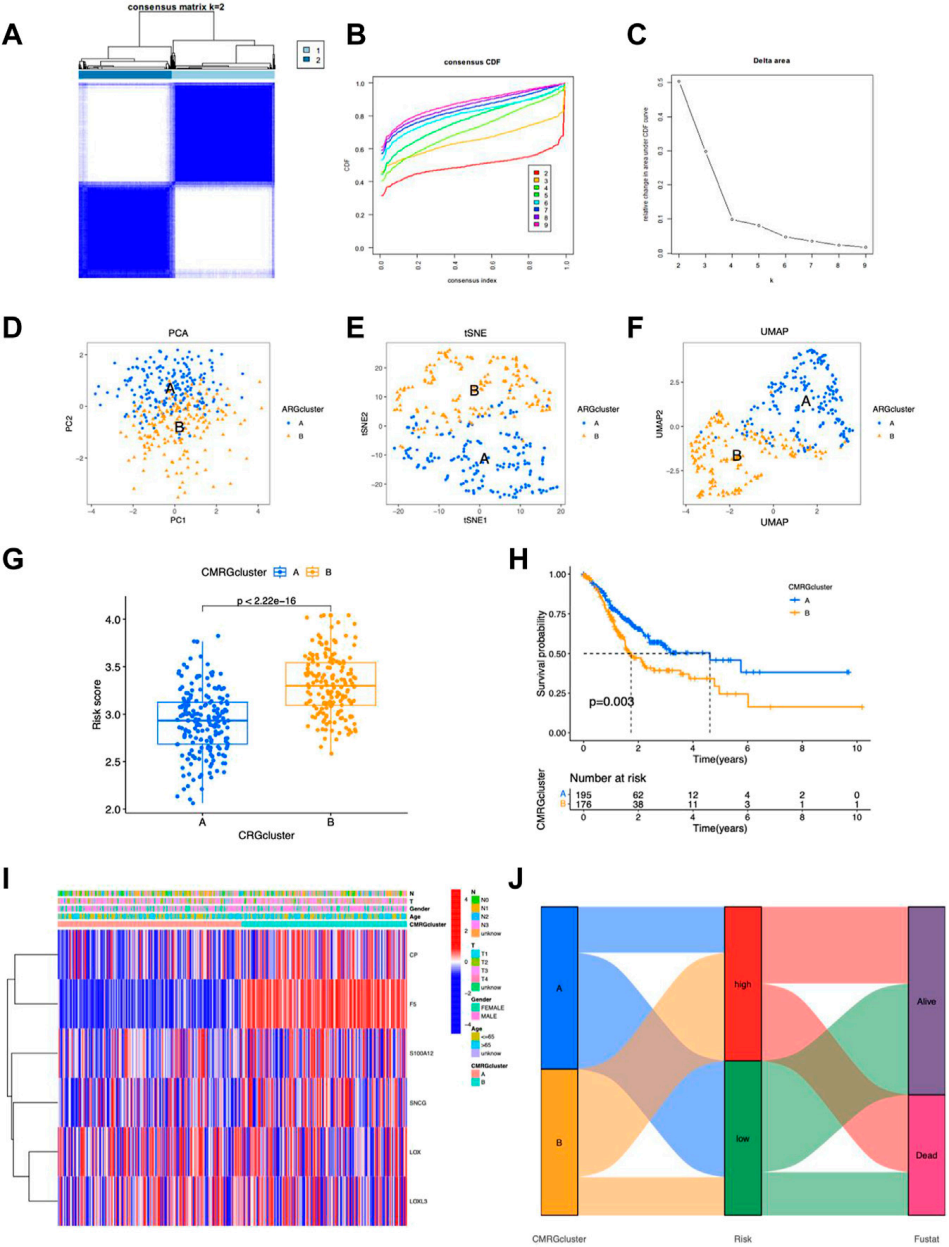
Characteristics of CMRG clusters. **(A)** Cluster plots for subtype analysis of the STAD sample. k = 2 for higher intra-group correlations but lower inter-group correlations. CDF **(B)** and delta plots **(C)** for consensus analysis. PCA analysis of two subgroups **(D)**, tSNE **(E)** and UMAP **(F)**. **(G)** Risk scores for the probability of survival for clusters A and **(B) (H)** Kaplan-Meier survival curves showing the probability of survival for clusters A and **(B) (I)** Heat map showing expression of clusters. **(I)** Heatmap of the 5 CMRGs expression in clinical features and clusters. **(J)** Sankey plots of CMRG clusters, risk scores, and survival.

The TCGA-STAD samples were clustered consistently according to the CMRG expression. In consensus clustering, the best clustering stability k = 2 was chosen (Figures 11A–C **)**. As a result, patients were divided into two subgroups with good resolution in the PCA (Figure 11D), tSNE (Figure 11E), and UMAP (Figure 11F) analyses. In addition, risk scores were higher in group B (Figure 11G) with a worse survival probability (*p* = 0.003) (Figure 11H), which provides preliminary evidence of the prognostic value of 5CMRG. The heat map depicts CMRGs expression and clinicopathological characteristics in the two groups. (Figure 11I). The connection for the subgroup, risk score, and survival are shown in Figure 11J.

### Validation of CMRGs

**FIGURE 12:**
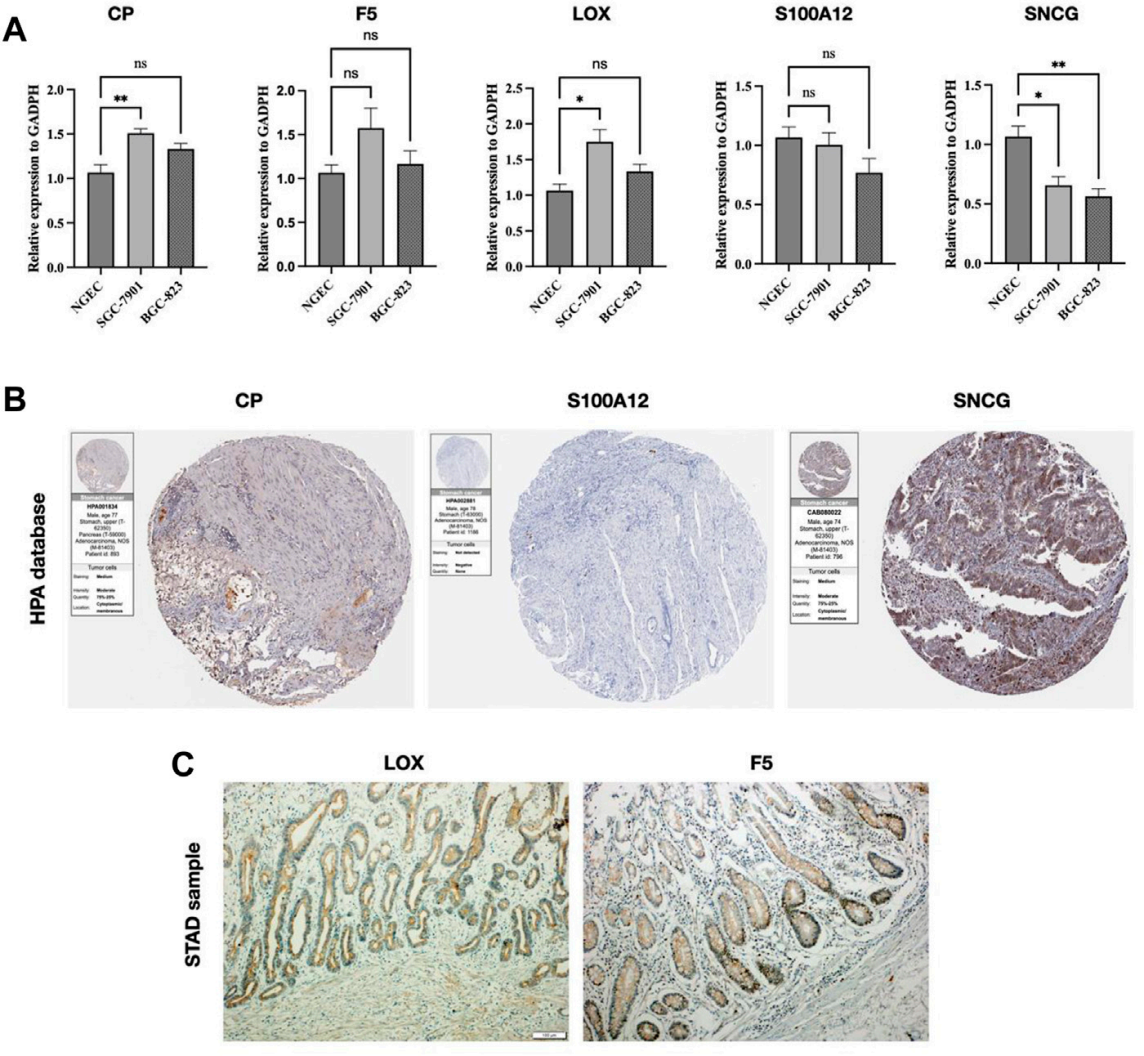
Validation of CMRG. **(A)** CMRG mRNA expression levels. **(B)** IHC analysis of CP, S100A12, and SNCG from the HPA database. **(C)** IHC analysis of LOX and F5 from STAD samples. *p < 0.05; **p < 0.01; ***p < 0.001.

We validated the mRNA level of the five CMRGs and found CP and LOX were increased in SGC-7901, and SNCG showed downregulated in SGC-7901 and BGC-823 (Figure 12A). Immunohistochemical results (per group, n = 3) for three hub CMRGs (CP, S100A12, and SNCG) from STAD patients were obtained from the HPA portal. According to the sample information of the database, the protein expression of CP and SNCG was higher in STAD samples, while S100A12 had no significant change (Figure 12B). There was no immunohistochemical data for the other 2 genes (LOX and F5) in HPA database. Hence, IHC analyses were performed on these two genes, and the examples of IHC staining of LOX and F5 were shown in Figure 12C. The expressions of LOX and F5 were found highly expressed in the mucosa of STAD tissues.

### Single-cell RNA-seq analysis

**FIGURE 13:**
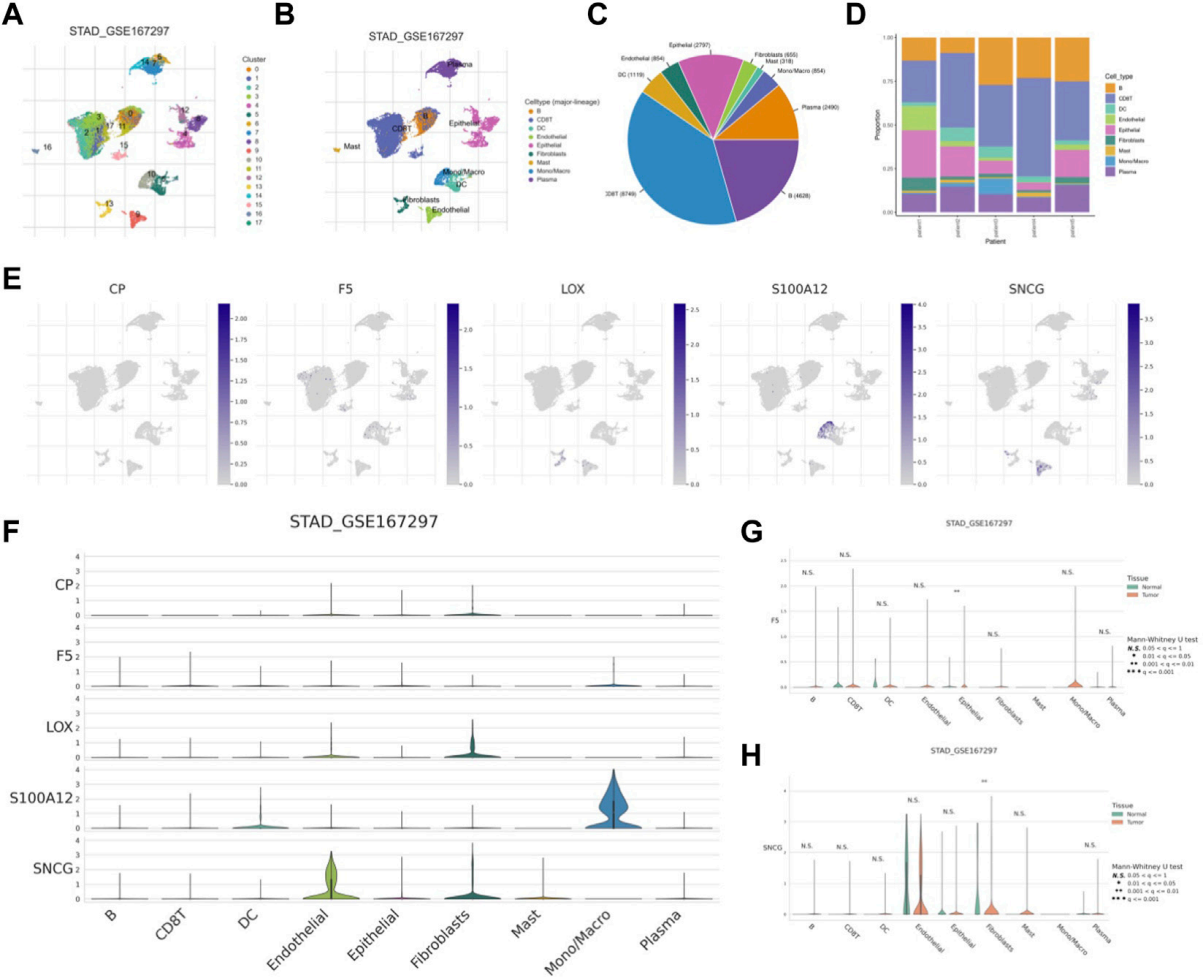
scRNA-seq analysis of CMRG. **(A)** Cells were divided into 17 clusters. **(B)** Annotation of the cell clusters. **(C, D)** The proportion of TICs in GSE167297. **(E)** Expression of CMRG mRNA in different TICs. **(F)** Distribution of CMRG in different cell types. Comparison of the expression levels of F5**(G)** and SNCG **(H)** in tumor and normal cells.

The UMAP approach was used to cluster the various TICs (Figures 13A, B). The proportions of TICs are shown in Figures 13C, D. Subsequently, higher expression levels of five CMRGs were detected in the respective clusters (Figure 13E), with S100A12 having a significantly higher average expression in the monocyte/macrocyte cluster; SNCG highly expressed (Figure 13F). In addition, F5 increased in tumor epithelial cells (Figure 13G); SNCG was significantly increased in fibroblasts from in tumor tissue (Figure 13H).

## Discussion

With the development of gastroscopy, the diagnosis of STAD has gradually increased, although patients diagnosed with STAD usually reached advanced stages (Smyth et al., 2020). As a result, surgery is ineffective, and the only treatment options available are chemotherapy, targeted therapy, and immunotherapy. As immunotherapeutic agents are being discovered and developed, they are increasingly challenging traditional treatment paradigms, such as chemotherapy and targeted agents. To better understand STAD, immunotherapy-associated genes are needed. This study identified copper metabolism-related genes (CMRG) as potential prognostic biomarkers for STAD. A risk model was subsequently established based on five CMRGs to elucidate the pathophysiology of STAD.

In the absence of a balanced copper supply, tumor growth leads to irreversible damage. High copper levels in serum and tissues are associated with the development of cancer. Copper can induce different types of cell death through several mechanisms, including apoptosis and autophagy, as well as oxidative stress, proteasome inhibition, and angiogenesis inhibition (Fukai et al., 2018; Shanbhag et al., 2021). Therefore, copper *in vivo* has attracted enormous attention and is a focus of research in the field of cancer therapy. Targeting copper is a novel strategy for cancer therapy (Li, 2020; Ruiz et al., 2021). The treatment of copper or proteins that metabolize copper has also been developed (Daniel et al., 2004). We identified five key STAD CMRGs, including CP, F5, LOX, S100A12, and SNCG, due to the important function of copper in tumorigenesis.

CP is known as Ceruloplasmin and is a serum iron-added enzyme. More than 95% of copper in plasma is transported by CP (Hellman and Gitlin, 2002). CP is a multifunctional molecule involved in iron metabolism and plays a role in cancer as it is involved in angiogenesis and neovascularization (Kunapuli et al., 1987). Invasive breast cancer and ICT infiltration have been associated with low expression of CP (Chen et al., 2021). Factor V (F5) is an important cofactor for blood coagulation and has been found associated with tumor aggressiveness (Tinholt et al., 2020). The epithelial-mesenchymal transition (EMT) is important for tumor growth. Overexpression of the EMT marker Lox was found in triple-negative breast tumors (Leo et al., 2018). γ-synuclein (SNCG) promotes metastasis of high-grade plasmacytoid ovarian cancer by acting on the PI3K/AKT signaling pathway (Zhang et al., 2020). Furthermore, scRNA studies revealed that SNCG was highly expressed in fibroblasts. CAFs are the most prominent component of tumors, and their interactions with tumor-infiltrating immune cells alter the antitumor immunological state in the TME (Rojas et al., 2020; The heterogeneity of cancer, 2023). Low expression of S100A12 can be used as a marker of tumorigenesis and progression in gastric cancer (Li et al., 2016). The ongoing interactions between tumor cells and the tumor microenvironment are critical in tumor genesis, development, metastasis, and therapeutic response (Xiao and Yu, 2021). Tumor-associated macrophages (TAMs) are one of the most common forms of tumor-infiltrating immune cells and are divided into two functionally distinct subtypes: classically activated M1 macrophages and alternatively activated M2 macrophages. M1 macrophages destroy tumor cells by directly mediating cytotoxicity and antibody-dependent cell-mediated cytotoxicity (Pan et al., 2020). TAM targeting is a growing subject of research, with the aim that these techniques may synergize with existing immunotherapies. The TME is very heterogeneous in terms of cellular composition, and the reciprocal connection between tumor epithelia and stromal cells influences cancer initiation and progression (Fang and Declerck, 2013). Furthermore, in many tumor forms, cancer-associated fibroblasts (CAFs) constitute the main cell type within the reactive stroma (Liao et al., 2019; The heterogeneity of cancer, 2023). Our results indicate the different expression profiles of CRMGs in the TICs, and the influence of these key genes on TICs and TMEs has never been elucidated, which necessitates further investigations.

STAD patients were categorized by the expression of the five CMRGs. Cox regression demonstrated the independence of the risk score. A nomogram was then created to facilitate clinical application, incorporating clinical features to provide a customized scoring system for physicians. A previous study showed that copper regulates key signaling pathways that underlie PD-L1-mediated immune evasion in cancer; reducing copper levels in tumor cells with copper chelators increases CD8^+^ T cells and inhibits cancer progression (Voli et al., 2020). Thus, we hypothesized that copper metabolism shuts down antitumor immunity. TIC and immune function were also different between the risk groups based on TME differences. M2 macrophages positively correlate with risk scores, and support tumor growth and distant metastases by collaborating in the anti-inflammatory response associated with tumor-associated macrophages (TAM) (Genin et al., 2015; Chen et al., 2017; Mehla and Singh, 2019; Rao et al., 2020; Yunna et al., 2020). This may explain why the prognosis for those with a high risk of developing cancer is poorer.

To better understand the mechanisms involved in copper metabolism, we performed GSEA and STAD on GSVA CMRGs. genes at low risk were enriched in the cell cycle pathway. As increased cell cycle activity suppresses anti-tumor immunity in cancer cells (Evan and Vousden, 2001; Li and Stanger, 2020), CMRGs may contribute to abnormal cell cycle regulation in STAD. In addition, immune pathways are enriched. Activation of the immune response requires copper function (Liu et al., 2022; Tulinska et al., 2022). Copper deficiency leads to decreased levels of interleukin-2, which results in T-cell dysfunction (Percival, 1998). The immunotherapy response was assessed by the IPS scores of the STAD and IMvigor210 cohort. Low-risk patients with TIME immune activation had a better prognosis and responded better to immunotherapy. In STAD patients with CMRG, immunotherapy can be guided more precisely. There has been evidence of a link between response to immunotherapy and genetic alterations (Jardim et al., 2021). The K-M plot also showed patients in the low TMB group with high risk had worse survival indicating an important role of TMB and risk score in tumor development.

The complexity of gastric cancer is largely determined by its molecular heterogeneity (Hu et al., 2021). A molecular subtyping technique can be very useful in predicting the occurrence and evolution of tumor polymorphisms, which leads to the exploration of better therapeutic interventions (De Re, 2018; Hu et al., 2021). It is essential to continuously explore and refine the STAD classification system to ensure it is fully valid and feasible. Our study determined the optimal cluster size (k = 2). By analyzing the PCA results, we confirmed the reliability of clustering. A STAD patient’s clinical outcome was significantly better in cluster A. Cluster B was notable for having a higher risk score, which could contribute to its better outcome. Differences in clinicopathology were found in both subgroups. Combined with these results, our study highlights the need for an updated STAD staging system. However, our study still has some shortcomings. To verify the risk model, multicenter investigations including *in vivo* experiments should be conducted. Furthermore, tumor immune microenvironments are exceedingly variable, and the risk model’s prediction capacity should be further examined.

## Conclusion

For STAD patients, a model for predicting immuno/chemotherapy response based on 5 CMRGs was created and validated. This model can help with prognostic prediction and can help facilitate the selection of appropriate treatment options for cancer patients.

## Data Availability

The original contributions presented in the study are included in the article/Supplementary Material, further inquiries can be directed to the corresponding author.

## Supplementary material

SUPPLEMENTARY FIGURE S1 Heatmap of differentially expressed CMRGs.

SUPPLEMENTARY FIGURE S2 Nomogram for PFS based on the CM.

SUPPLEMENTARY FIGURE S3 GSVA for CMRG.

SUPPLEMENTARY FIGURE S4 The CNV frequency analysis.
