# Supplementary figures and images for: Development of a novel copper metabolism-related risk model to predict prognosis and tumor microenvironment of patients with stomach adenocarcinoma
Source: Front Pharmacol. 2023 May 22;14:1185418. doi: 10.3389/fphar.2023.1185418 (PMC10241246; doi:10.3389/fphar.2023.1185418)

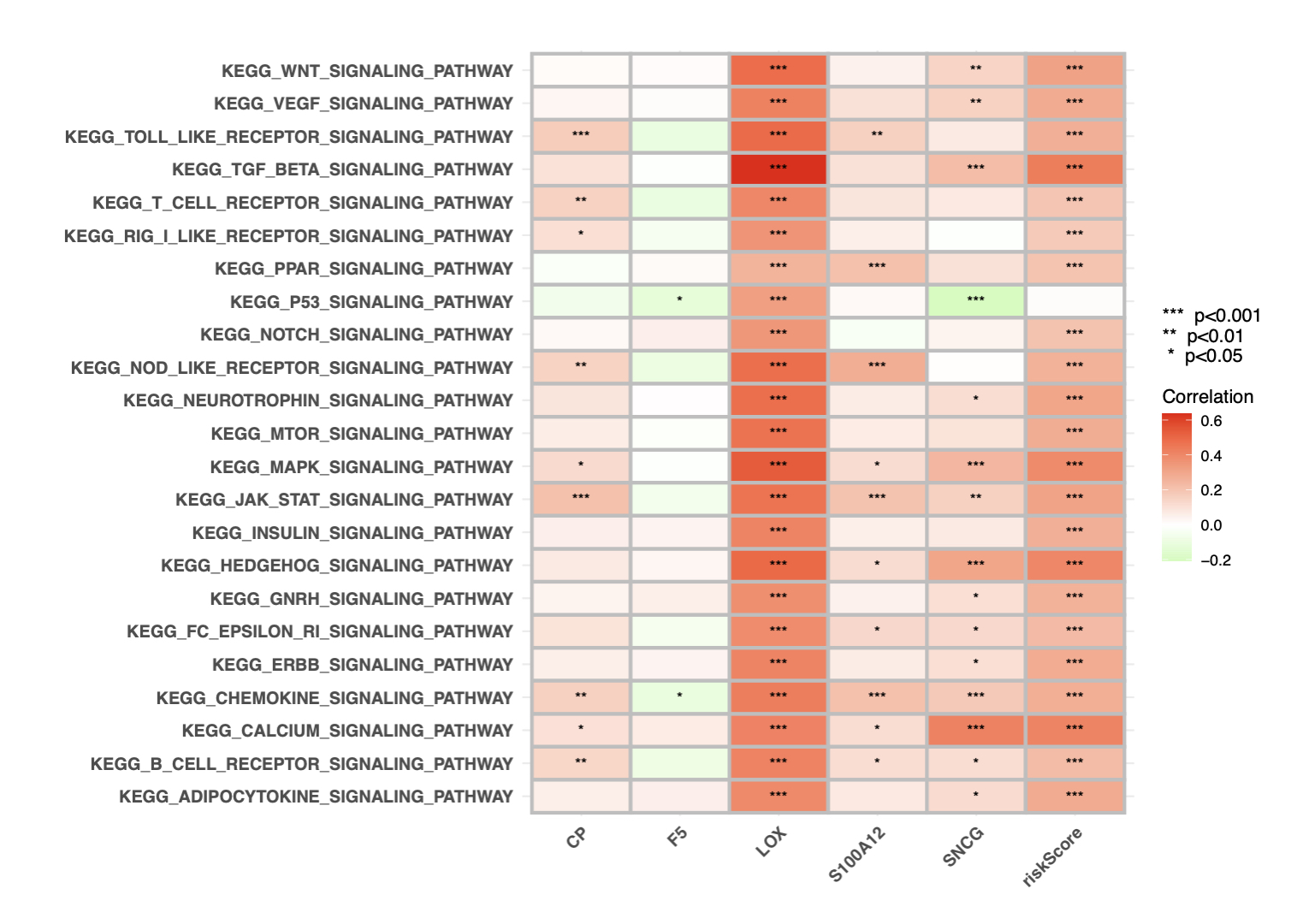

Supplement: Supplementary file 2 [file Image3.JPEG]

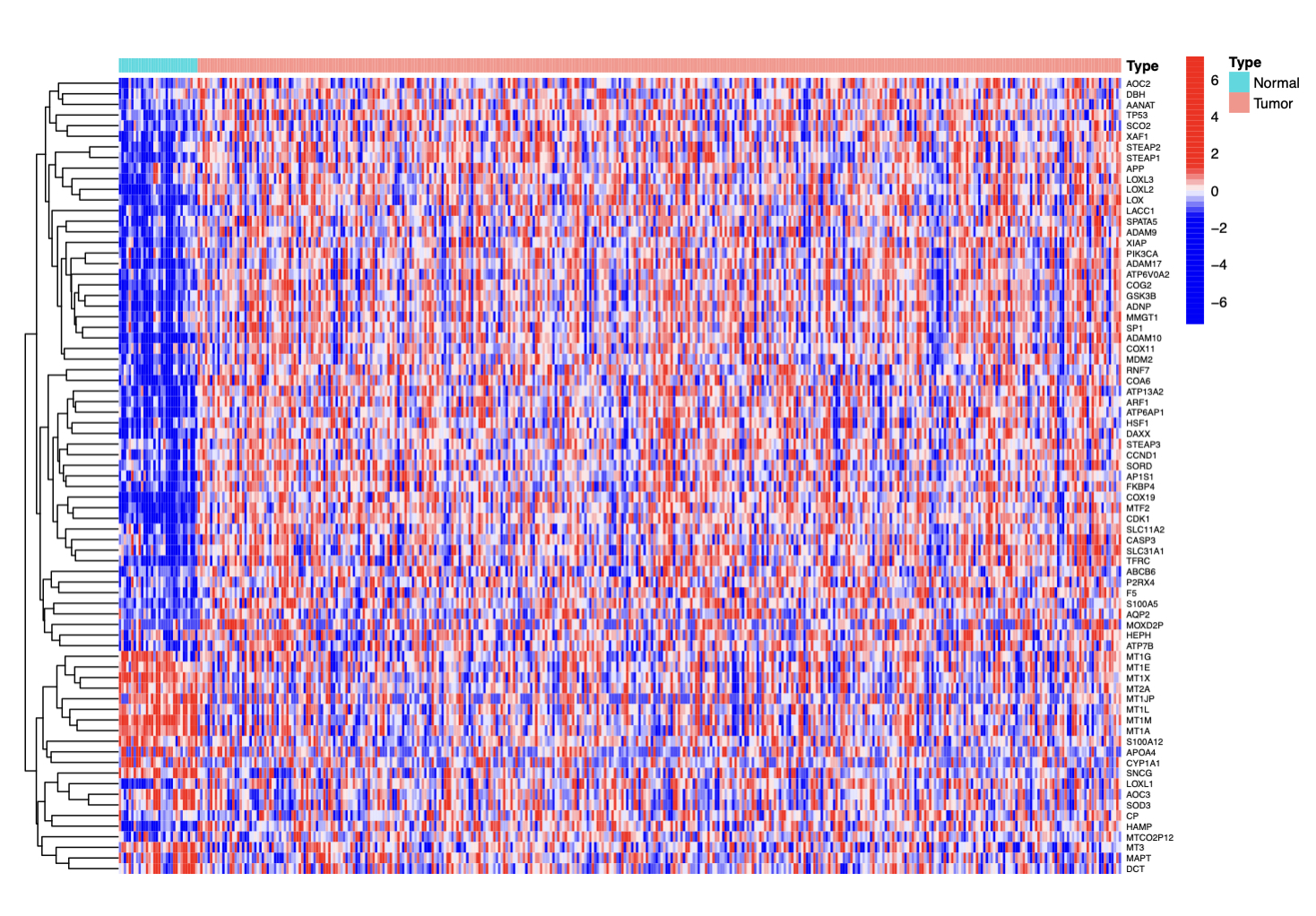

Supplement: Supplementary file 3 [file Image1.JPEG]

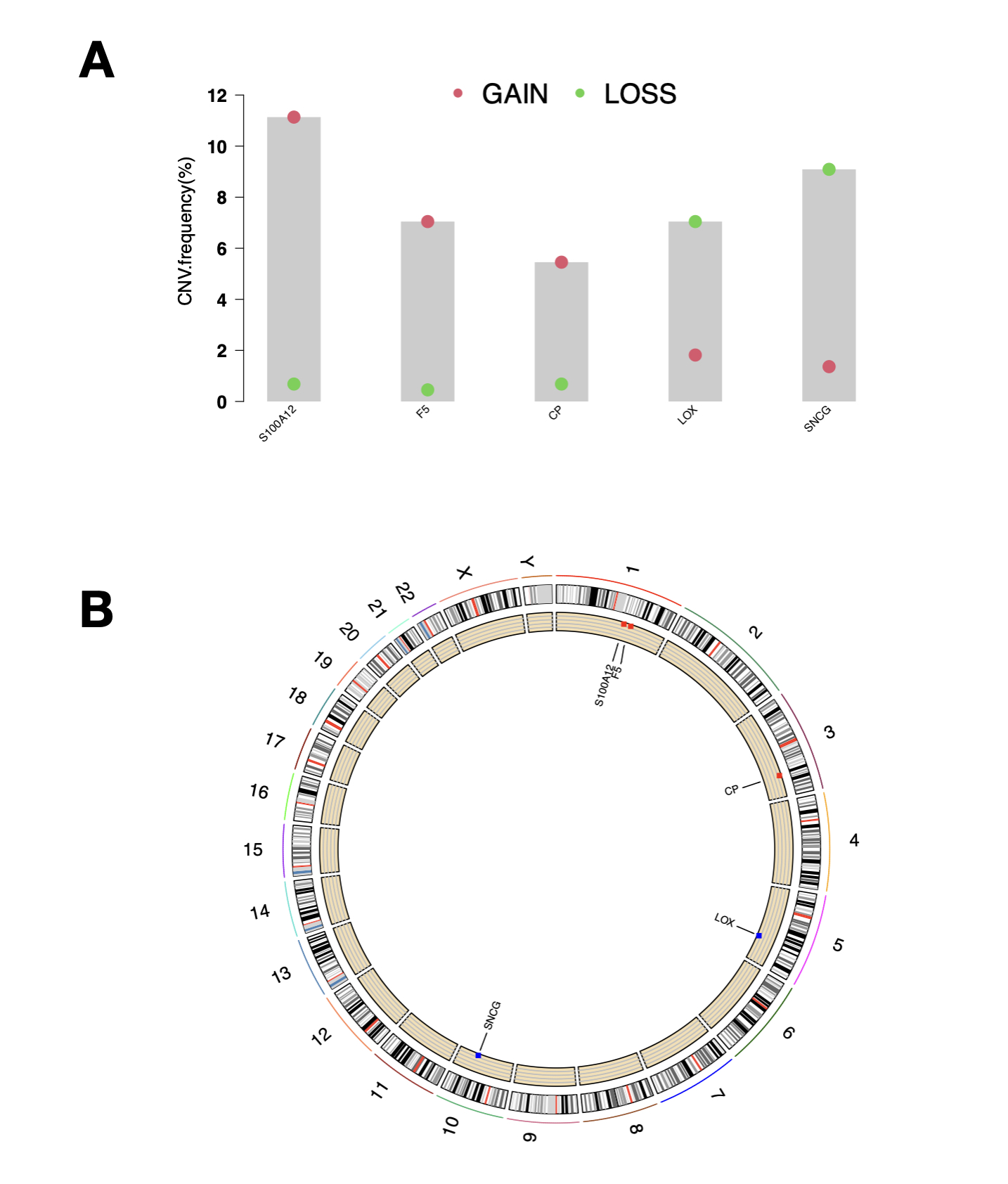

Supplement: Supplementary file 4 [file Image4.JPEG]

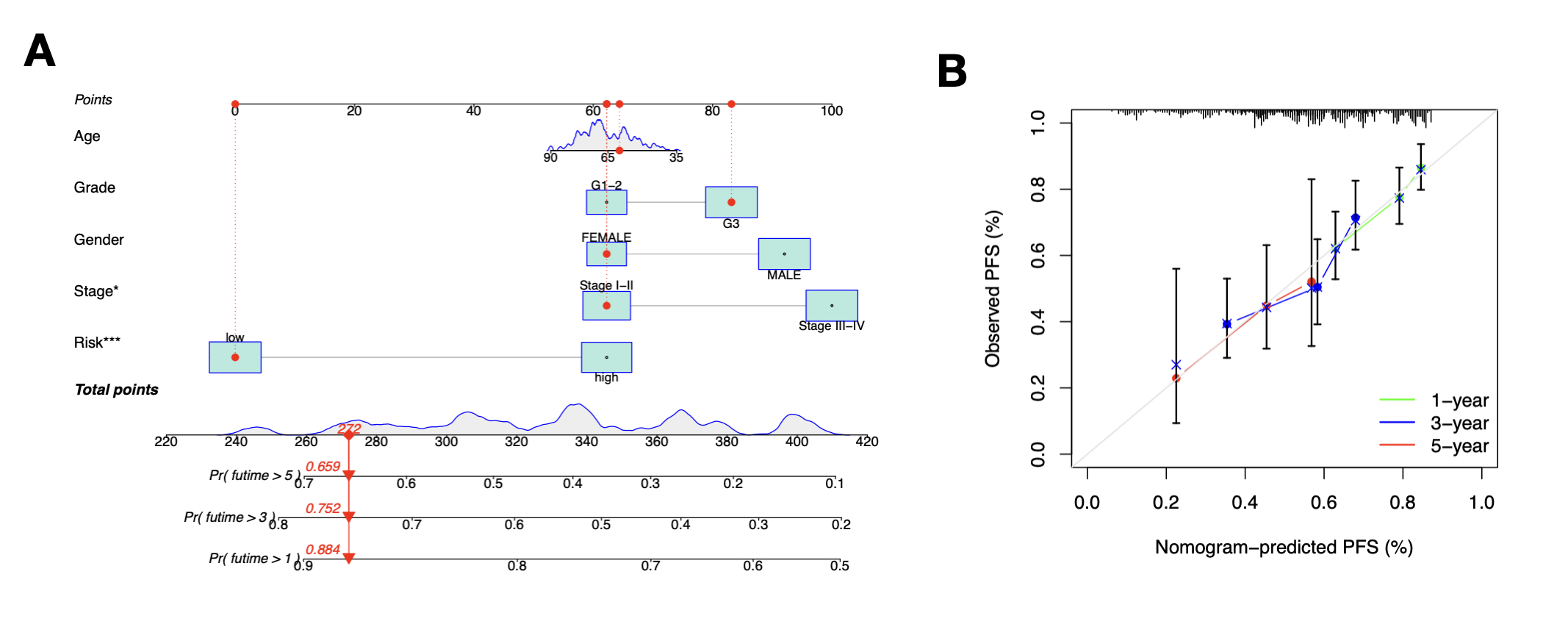

Supplement: Supplementary file 5 [file Image2.JPEG]
